# The Drop of Allergen Immunotherapy for Respiratory Allergy in Italy: An Assessment of Sales

**DOI:** 10.1111/cea.70183

**Published:** 2025-12-04

**Authors:** Mattia Giovannini, Antonio Bognanni, Simona Barbaglia, Luisa Brussino, Cristiano Caruso, Francesco Catamerò, Domenico Gargano, Enrico Heffler, Giovanni Paoletti, Giorgio Walter Canonica

**Affiliations:** ^1^ Department of Health Sciences University of Florence Florence Italy; ^2^ Allergy Unit Meyer Children's Hospital IRCCS Florence Italy; ^3^ Clinical and Epidemiology and Research Center (CERC), Department of Biomedical Sciences, IRCCS Humanitas Research Hospital Humanitas University Milan Italy; ^4^ Department of Biomedical Science Humanitas University Milan Italy; ^5^ Personalized Medicine, Asthma and Allergy IRCCS Humanitas Research Hospital Milan Italy; ^6^ Associazione Nazionale Pazienti ‘Respiriamo Insieme‐APS’ Padua Italy; ^7^ Department of Medical Sciences University of Turin Turin Italy; ^8^ Immunology and Allergy Unit, AO Ordine Mauriziano di Torino Turin Italy; ^9^ UOSD Allergy and Clinical Immunology Unit, Fondazione Policlinico A. Gemelli IRCCS Rome Italy; ^10^ Catholic University of Sacre Heart Rome Italy; ^11^ Allergy and Immunology Unit, Azienda Ospedaliera S. Giuseppe Moscati Avellino Italy

**Keywords:** allergen immunotherapy, allergy, Italy, market analysis, sales trend

## Abstract

- Allergen immunotherapy sales in Italy experienced a sharp decline between 2008 and 2016, followed by a more gradual decrease through 2023.
- Differences in reimbursement, along with structural, educational, and regulatory factors, likely contributed to the decline, and warrant further research.

To the Editor,

Allergen immunotherapy (AIT) remains the only treatment capable of modifying the natural course of allergies [1, 2]. The literature highlights its benefits, including a reduction in work and school absences and favourable patient‐reported safety and efficacy profiles for respiratory allergy [3, 4, 5, 6]. However, despite these advantages, a general lack of clarity and awareness regarding AIT's applications persists, leading to potential misinformation or misuse [3, 5]. To advance the understanding in this field, we aimed to evaluate sales trends of AIT for respiratory allergy in Italy.

This assessment was promoted and conducted by the national patient association ‘Respiriamo Insieme‐APS’ in collaboration with its Scientific Committee and the authors of the article. The association, founded in 2014 and registered in ‘Registro Unico Nazionale del Terzo Settore’, has approximately 3500 members, including patients with respiratory disorders, caregivers, family members, and specialists such as allergists, anthropologists, paediatricians, pulmonologists, and psychologists.

‘Respiriamo Insieme‐APS’ collected anonymous data on AIT sales (product units) from a panel of eight companies operating in the Italian market between 2008 and 2023 through CERVED. The company panel composition varied over time due to market exits, acquisitions, and re‐entries. Specifically, in 2010, one company exited the panel but rejoined in 2013; in 2016, one company was acquired by another; and in 2020, one company permanently left the market. Aggregated data were provided by companies, without the possibility of deepening their granularity, for example, additional characteristics or patients treated.

Our evaluation was approved by the Ethics Committee ‘Campania Nord’ (registry CECN/2098, 26‐apr‐2023). The collected data were analysed using STATA/MP 16.1 (College Station, TX, USA: Stata Corp LP). We did not perform inferential analyses to generalise findings to a broader population due to the deterministic nature of our dataset. Instead, we conducted descriptive analyses and used linear regression to quantify the linear reduction in sales and identify potential timepoints of sales variations.

The results showed a marked decline in AIT sales in Italy, with an annual reduction of 17334 units. A linear trend assessment identified 2016 as a potential turning point in this decline's rate. A Chow Test confirmed a structural break in 2016, describing a 63.35% sales drop between 2008 and 2016 (−27,719 units per year) and a slower but steady 30.32% decline from 2016 to 2023 (−6707 units per year). Given this shift, we focused on the period up to 2016 to better understand the factors contributing to the steeper decline phase.

The sales drop was observed across all AIT product categories. Injectable therapies declined by 59%, with a sharper decrease in induction therapy (68%) compared to maintenance (42%). Similarly, non‐injectable therapy sales declined by 64%, with maintenance therapy showing the most pronounced drop (76%) compared to induction (60%).

To explore whether reimbursement policies influenced these trends, we examined regional sales patterns. In Lombardy and Piedmont, where AIT is respectively fully and partially reimbursed by the national healthcare system, sales declined by 54% and 68%. However, the similarity with the sales decline in other regions combined (66%) suggests that reimbursement policies alone do not fully explain this trend (Figure 1A–D).

**FIGURE 1:**
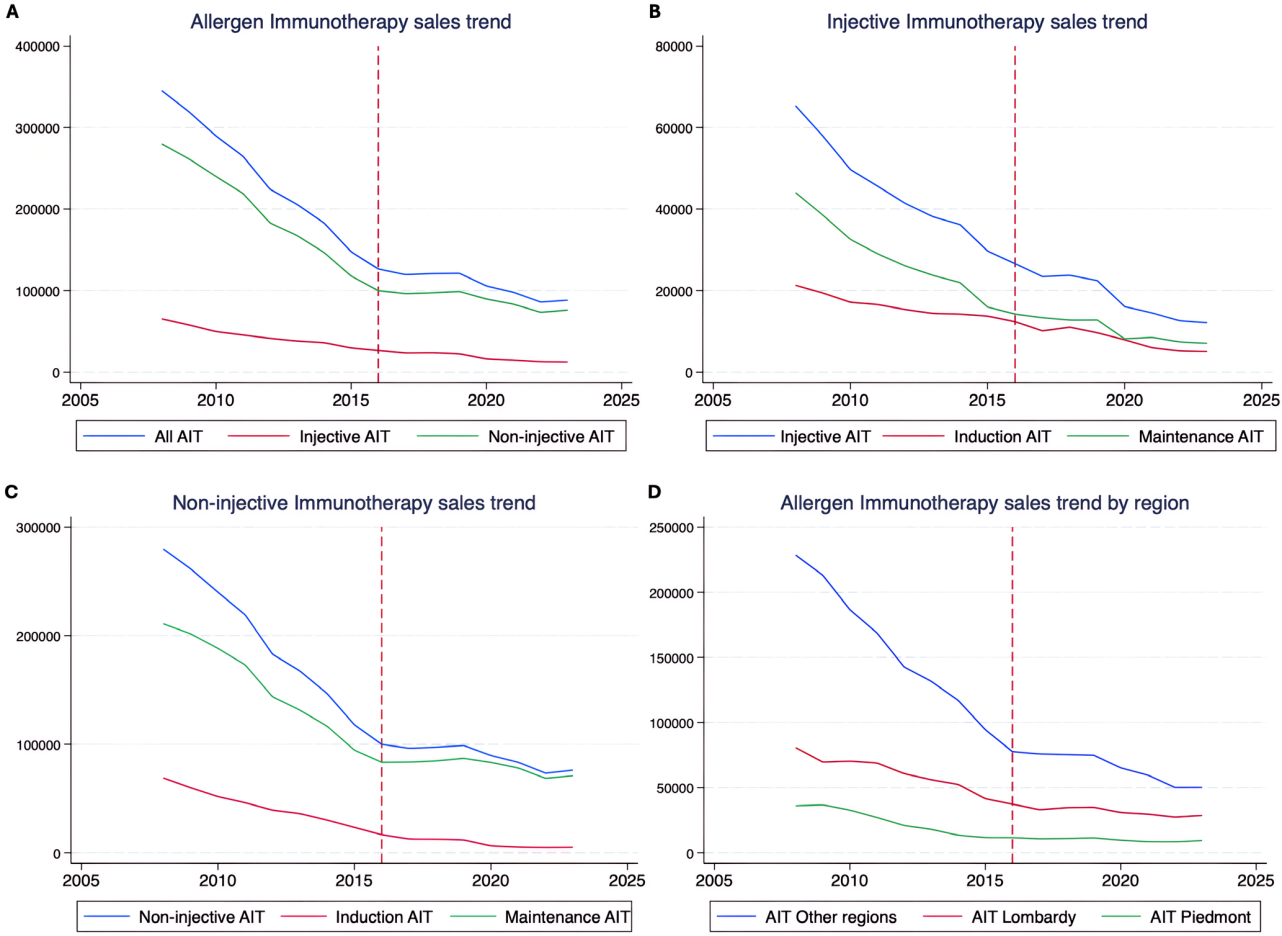
Linear sales trend per year of (A) total AIT sales—total reduction: 63.35%; injective therapies: 59.27% decrease; non‐injective therapies: 64.30% decrease; (B) sales of injective AIT—total injective therapy: 59.27% decrease; maintenance injective therapy: 67.67% decrease; induction injective therapy: 41.91% decrease; (C) sales of non‐injective AIT—total non‐injective therapy: 64.30% decrease; maintenance non‐injective therapy: 60.50% decrease; induction non‐injective therapy: 75.92% decrease; (D) total AIT sales volume by region—total reduction (excluding Lombardy and Piedmont): 66.03%; total reduction in Lombardy: 53.73%; total reduction in Piedmont: 67.87%.

Given the multi‐factorial nature of this phenomenon, we propose several co‐factors that may have contributed to the decline in AIT sales and should be further assessed in future research. A potential decline in structured training programs may have resulted in fewer specialists being equipped to prescribe AIT appropriately. Hence, the number of specialised centres with expertise in prescribing AIT has possibly decreased, which may have provoked a further restriction to patients' access to these therapies.

Our assessment has several limitations, including its retrospective design and the exclusion of AIT products registered as drugs, not collected through CERVED. However, among its strengths, this represents the first evaluation of AIT sales trends in the Italian context, and it benefits from a comprehensive dataset, providing valuable insights into long‐term sales patterns.

Although the sales decline has slowed post‐2016, several barriers continue to hinder AIT adoption. Moving forward, establishing a unified national regulatory framework with clear pathways appears essential to restoring confidence among interest‐holders. In parallel, enhancing physician education and training seems crucial to ensuring appropriate AIT prescription.

Strengthening both regulatory consistency and clinical expertise will help position AIT as a key component of allergy management, shifting the focus from signs and symptoms control to immunomodulation, with the potential to reduce the burden of allergic diseases and improve long‐term outcomes for respiratory allergy. Finally, we support more suitable financial accessibility through national‐level standardisation in therapy refundability. Importantly, the partnership with the patient association played a pivotal role, without which this research would have been difficult to accomplish.

## Author Contributions

All authors approved the final version of the manuscript as submitted and agreed to be accountable for all aspects of the work.

## Funding

This research did not receive any specific grant from public, commercial, or non‐profit funding agencies.

## Ethics Statement

The study protocol was approved by the Ethics Committee ‘Campania Nord’ (registry CECN/2098, 26‐Apr‐2023).

## Conflicts of Interest

M.G. reports personal fees from Sanofi, Thermo Fisher Scientific. G.P. reports fees for speaker activities and/or advisory boards participation from Lofarma, GSK, and AstraZeneca, outside the submitted work. G.W.C. reports research or clinical trials grants paid to his institution from Menarini, AstraZeneca, GSK, Sanofi Genzyme and fees for lectures or advisory board participation from Menarini, AstraZeneca, CellTrion, Chiesi, Faes Farma, Firma, Genentech, Guidotti‐Malesci, GSK, HAL Allergy, Innovacaremd, Novartis, OM‐Pharma, Red Maple, Sanofi‐Aventis, Sanofi‐Genzyme, Stallergenes‐Greer and Uriach Pharma, outside the submitted work. E.H. reports fees for speaker activities and/or advisory boards participation from Sanofi, Regeneron, GSK, Novartis, AstraZeneca, Stallergenes‐Greer, Chiesi, Almirall, Bosch, Lofarma, outside the submitted work. All other authors report no conflicts of interest related to this work.

## Data Availability

The data that support the findings of this study are available from CERVED. Restrictions apply to the availability of these data, which were used under licence for this study. Data are available from the author(s) with the permission of CERVED.
